# *Sedeveria pink ruby* Extract-Mediated Synthesis of Gold and Silver Nanoparticles and Their Bioactivity against Livestock Pathogens and in Different Cell Lines

**DOI:** 10.3390/antibiotics12030507

**Published:** 2023-03-03

**Authors:** Palaniselvam Kuppusamy, Sujung Kim, Sung-Jo Kim, Myunghum Park, Ki-Duk Song

**Affiliations:** 1 Department of Agricultural Convergence Technology, Jeonbuk National University, Jeonju 54896, Republic of Korea; 2 Division of Cosmetics and Biotechnology, College of Life and Health Sciences, Hoseo University, Asan 31499, Republic of Korea; 3 Research and Development Center, T&T Research, Anyang 14059, Republic of Korea

**Keywords:** *S. pink ruby*, SP-AuNPs, SP-AgNPs, FESEM-EDX, antimicrobial, cytotoxicity

## Abstract

Biological synthesis of metal nanoparticles has a significant impact in developing sustainable technologies for human, animal, and environmental safety. In this study, we synthesized gold and silver nanoparticles (NPs) using *Sedeveria pink ruby* (SP) extract and characterized them using UV–visible spectrophotometry, FESEM-EDX, HR-TEM, XRD, and FT-IR spectroscopy. Furthermore, antimicrobial and antioxidant activities and cytotoxicity of the synthesized NPs were evaluated. UV–visible absorption spectra showed λ_max_ at 531 and 410 nm, corresponding to the presence of SP gold NPs (SP-AuNPs) and SP silver NPs (SP-AgNPs). Most NPs were spherical and a few were triangular rods, measuring 5–30 and 10–40 nm, respectively. EDX elemental composition analysis revealed that SP-AuNPs and SP-AgNPs accounted for >60% and 30% of NPs, respectively. Additionally, some organic moieties were present, likely derived from various metabolites in the natural plant extract, which acted as stabilizing and reducing agents. Next, the antimicrobial activity of the NPs against pathogenic microbes was tested. SP-AgNPs showed potent antibacterial activity against *Escherichia coli* and *Yersinia pseudotuberculosis*. Moreover, at moderate and low concentrations, both NPs exhibited weak cytotoxicity in chicken fibroblasts (DF-1) and macrophages (HD11) as well as human intestinal cancer cells (HT-29). Meanwhile, at high concentrations, the NPs exhibited strong cytotoxicity in both chicken and human cell lines. Therefore, the synthesized SP-AuNPs and SP-AgNPs may act as promising materials to treat poultry diseases.

## 1. Introduction

Nanotechnology is advanced science with broad applicability in different areas, such as chemistry, physics, mechatronics, biotechnology, microbiology, and environmental sciences [1,2]. Nanoparticles (NPs) possess attractive qualities useful for the development of nano-biosensors, probes, therapeutic agents, vaccine adjuvants, drug delivery vehicles, and toxin removal systems [3,4]. NPs can be synthesized via conventional physical and chemical methods, albeit with certain limitations, primarily the use of toxic reagents in synthesis steps to prevent aggregation and the requirement of severe reaction conditions. In addition, the conventional methods of NP synthesis show side effects, poor solubility, undesirable activity in therapeutic interventions, and environmental hazards. Moreover, the chemical procedures may produce certain unwanted by-products and/or unreacted substances, which remain in the synthesized colloidal medium, rendering it unsuitable for medical applications [5,6]. Therefore, green chemistry approaches have been developed to synthesize stable monodispersed NPs with low toxicity. Green chemistry-based synthesis approaches use a wide range of biological resources, such as plant extracts, bacteria, fungi, microalgae, and seaweeds, to obtain nanosized materials [7,8]. These materials are low-cost, eco-friendly, sustainable, and suitable for scaled-up production [9,10,11]. Nonetheless, some drawbacks of green NP production must be acknowledged; the most obvious shortcoming is that these procedures do not guarantee materials of a consistent shape and size. The size and morphology of nanomaterials significantly impact their applications [12,13,14]. NPs are classified into two major classes, namely (1) inorganic NPs, such as metal NPs (Au, Ag, Cu, and Al), magnetic NPs (Co, Fe, and Ni), and semiconductor NPs (ZnO, ZnS, and CdS), and (2) organic NPs, such as carbon-based materials, quantum dots, carbon nanotubes, and silicon oxide nanotubes. Gold NPs (AuNPs) possess many valuable functional properties, such as optical and electrochemical characteristics, and show a high affinity for molecules of various molecular weights, such as RNA, DNA, and proteins. The optical properties of AuNPs, expressed as specific λ_max_ values, have prompted their use in various biomedical and clinical applications, such as biosensors, gene therapy, drug delivery, bioinstrumentation, and bio-imaging techniques [15,16]. Likewise, silver NPs (AgNPs) are noble materials primarily composed of silver oxide [17]. AgNPs present electrical and catalytic properties, large surface area, and chemical reactivity, allowing them to bind with various ligands. AgNPs have been widely used to fabricate an array of commercial products, including surgical instruments, food storage containers, textile coatings, surgical masks, filters, toothpaste, tissue scaffold materials, and other medical accessories [18,19].

Furthermore, plant bioactive metabolites can be employed to synthesize NPs and control agglomeration during NP synthesis [20,21,22,23]. However, biologically derived NPs have certain limitations, such as the lack of standardization synthesis procedures, fluctuating concentrations of phytochemicals in plant extracts, effects of geography and climate on plant sources, difficulties in predicting the major compounds emerging from synthesis steps, and challenges in identifying the binding of targeted phytochemicals on the surface of NPs. However, synthesis procedures can be optimized by modulating the synthesis reaction parameters, such as pH, temperature range, metal salt concentration, and reaction time. Such optimized reaction conditions may improve the properties of biosynthesized NPs and promote the formation of NPs with a desired size and shape [24,25,26]. Recently, *Ferocactus echidne* was shown to rapidly reduce silver ions into nano-silver without the use of any external chemical agent; moreover, the cactus extract-stimulated NPs were active against Gram-positive and Gram-negative bacteria and fungi (e.g., *Candida albicans*), indicating the potential use of the synthesized NPs as potent broad-spectrum antibiotic and antifungal agents [27]. In addition, these NPs may be used as novel antimicrobial agents and food additives to improve the health of poultry and other livestock animals.

**Figure 1:**
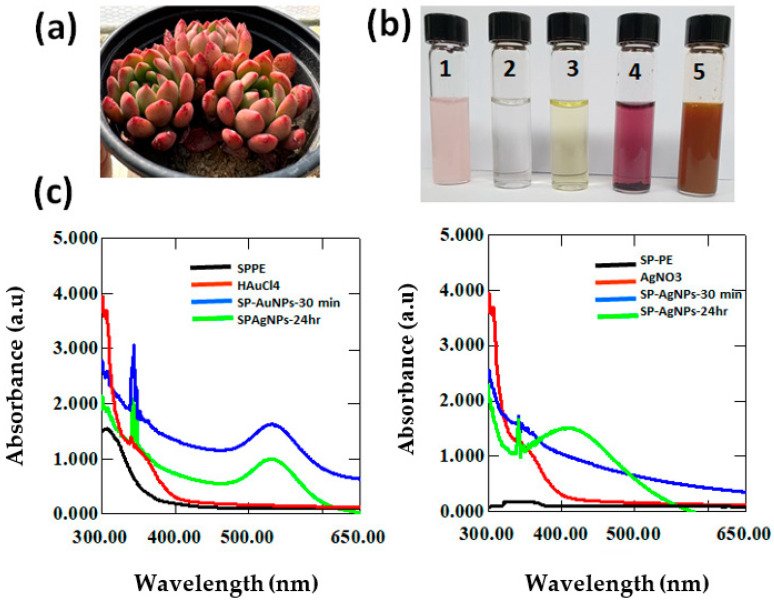
UV–Vis spectra of SP-mediated AuNP and AgNP colloidal solution measured at different time intervals (30 min over 24 h). (**a**) Photographs of *Sedeveria pink ruby* plant. (**b**) Colors of solutions indicating the redox reaction ((1) SP aqueous plant extract, (2) AgNO_3_, (3) HAuCl_4_, (4) SP-AuNPs, and (5) SP−AgNPs). (**c**) UV–Vis spectra of AuNPs and AgNPs exhibiting λ_max_ at 531 and 410 nm, respectively.

*Sedeveria pink ruby* is an ornamental succulent plant (Figure 1a). It is a cross species of *Sedum* and *Scheveria* succulents. *S. pink ruby* is rosette-shaped with thick padded leaves. It can grow in conditions of low water and high temperature. However, the bioactivities of *S. pink ruby* and its bioactive constituents have not been reported in the literature. To the best of our knowledge, the present study is the first to report the biological synthesis of AuNPs and AgNPs using the aqueous extract of *S. pink ruby* and study their biological properties. Briefly, we synthesized AuNPs and AgNPs using *S. pink ruby* aqueous extract (SP-AuNPs and SP-AgNPs, respectively) and characterized them using spectroscopic and electron microscopic techniques. Furthermore, we examined the antimicrobial effects of the synthesized SP-AuNPs and SP-AgNPs against livestock pathogens, their antioxidant activity, and their cell cytotoxicity using various chicken and human cell lines.

## 2. Results and Discussion

### 2.1. UV–Visible (UV–Vis) Spectrophotometry of SP-AuNPs and SP-AgNPs

AuNPs and AgNPs were biofabricated from metal salt precursors HAuCl_4_ and AgNO_3_, respectively, using *S. pink ruby* aqueous extract. NP formation was initially confirmed based on color changes in the reaction solution after mixture, as the color gradually intensified. The presence of SP-mediated AuNPs was apparent as there was an immediate change to pale purple after the addition of plant extract. Similarly, upon the addition of plant extract to silver nitrate solution, the color changed to yellowish-brown within 20 min due to the reduction of silver ions to AgNPs (Figure 1b). Both NP mixtures were subjected to UV–Vis spectrophotometry at specific time intervals (30 min for 24 h). Figure 1c shows the absorption spectra of the synthesized AuNPs and AgNPs, with λ_max_ at 531 and 410 nm, respectively.

Furthermore, metal NP production was optimized, with enhanced yield and reduced production cost, via green synthesis. In the present study, we used three major key factors, namely pH, plant extract ratio, and metal ion concentration, to regulate and optimize NP synthesis. As shown in Figure S1a–f, an increase in pH affected the reduction time and color of the reaction mixture, reflecting changes in the size and shape of NPs. Moreover, precipitation increased at lower pH values in both SP-AuNPs and SP-AgNPs (Figure S1a,d). Furthermore, a higher metal ion concentration in the synthesis medium resulted in a broader UV–Vis absorption peak, slower reduction rate, and gradual appearance of color in the colloidal medium. However, the addition of plant extracts rapidly converted the metal ions (Au^3+^ and Ag^+^) into AuNPs and AgNPs. However, a 1:2 ratio of plant extracts to metal ions reduced metal ions more efficiently than a higher or lower ratio. Of note, the UV–Vis spectra became sharper, and the optical density increased slightly, indicating that the NPs showed a distinct size and shape. The appearance of a pale pink, brownish color in the nanosolution suggests the reduction of metal ions and formation of NPs and reflects the related surface plasmon resonance of AuNPs and AgNPs in the UV–Vis range. The peaks of SP-AuNPs and SP-AgNPs were within the previously reported wavelength ranges for metal NPs synthesized from with various plant extracts [28,29,30]. Similarly, in a previous study using *Clerodendrum inerme* (CI) leaf extract, the absorption maximum was detected at 534 nm for Cl-AuNPs and at 412 nm for Cl-AgNPs. In addition, the CI leaf extract exhibited a strong absorption band at 380 nm, suggesting the contributions of polyphenolics and flavonoids to NP synthesis [31]. For optimum synthesis, the core plant extract and water dilution ratio of 1:1 was economic, and the peak for Cl-AuNPs biosynthesized from the CI extract overlapped with that for AuNPs produced via biological approaches. For, AgNP synthesis, plant extract without dilution was optimal. The optimized gold and silver salt concentration was 2–5 mM; at lower or higher salt concentrations than this range, NP yield decreased and peaks broadened; in other words, particle stability decreased, leading to agglomeration [32,33]. pH plays a crucial role in NP synthesis, as the stability of the synthesis medium can change due to alteration of the nature of bioactive compounds present in it. Changes in pH may reduce the capping ability of molecules and prevent the subsequent growth of NPs [34].

### 2.2. FESEM-EDX Characterization of SP-AuNPs and SP-AgNPs

**Figure 2:**
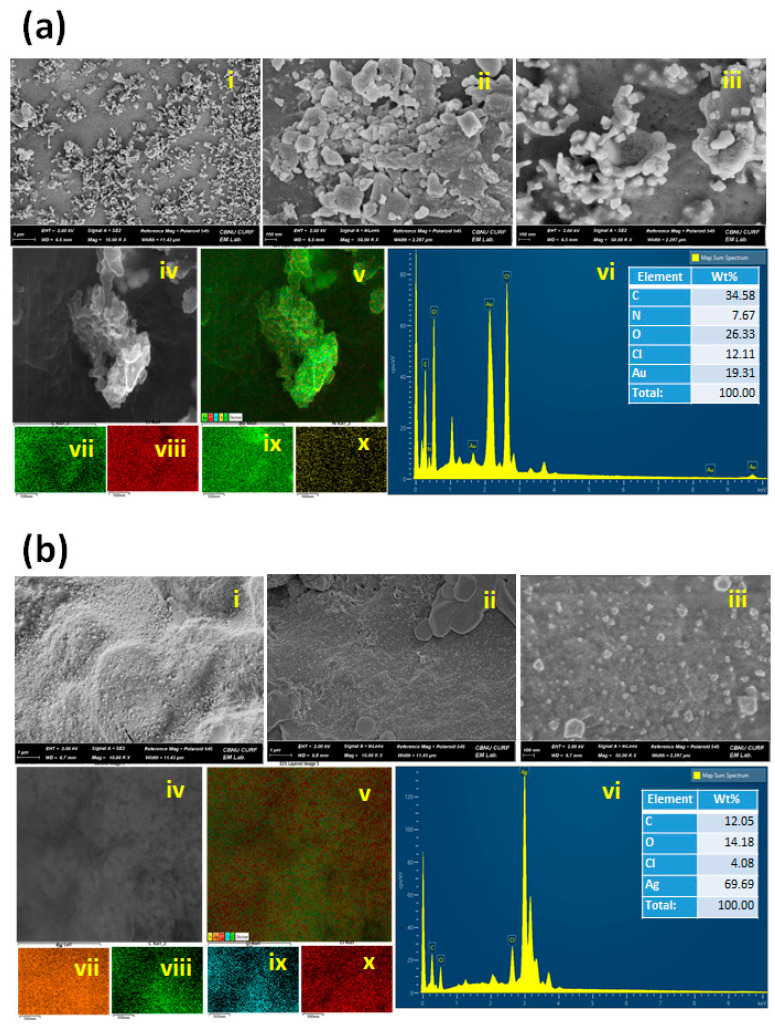
Field-emission scanning electron micrograph of SP-synthesized AuNPs and AgNPs. (**a**) (**i**–**iii**) Size and morphology of the synthesized AuNPs; scale bar = 100 nm. (**iv**–**ix**) Elemental mapping for purity and distribution analysis in the selected electron micrograph region of the nano-gold sample. (**x**) Energy-dispersive X-ray spectrum of synthesized AuNPs (inset table presents element ratios in wt%). (**b**) (**i**–**iii**) Size and morphology of the synthesized AgNPs; scale bar = 100 nm. (**iv**–**ix**) Elemental mapping for purity and distribution analysis in the selected electron micrograph region of the nano-silver sample. (**x**) Energy-dispersive X-ray spectrum of synthesized AgNPs (inset table presents element ratios in wt%).

Figure 2a,b show the results of an FESEM-EDX analysis of the size and morphology of SP-AuNPs and SP-AgNPs. SP-AuNPs existed in monodispersed spherical forms, although some irregular particles were observed in the size range of 5–30 nm. Similarly, SP-AgNPs were present in monodispersed spherical forms ranging in size from 10 to 40 nm. These results are consistent with previous reports [35] of spherical NPs synthesized from *Catharanthus roseus* leaf extract ranging in size from 35 to 50 nm.

The energy-dispersive X-ray spectroscopy (EDX) spectra of SP-AuNPs and SP-AgNPs are shown in Figure 2a(x),b(x). Strong elemental signals were detected at 3 and 2.4 keV, corresponding to the presence of gold and silver metals, respectively. Weaker signals corresponding to sodium, carbon, and oxygen were identified, which may be derived from certain biomolecules in aqueous SP extract. Furthermore, we explored the elemental mapping of SP-AuNPs, which consist primarily of gold, carbon, sodium, copper and oxygen (Figure 2a(iv–ix)). A large proportion of gold (~20%) was identified, with strong characteristic spots on the selected area of NPs. Lower proportions of carbon, hydrogen, copper, and oxygen were confirmed at respectively ~15%, ~12%, ~10%, and ~7% in the samples. The appearance of elements such as carbon, copper, and oxygen may partly be attributed to the use of plant extract and a carbon conductive film for microscopic sample preparation. In SP-AgNPs, silver metal accounted for 70%, while carbon (25%), oxygen (20%), and copper (5%) were detected at smaller proportions (Figure 2b(iv–ix)). Nevertheless, the proportion of gold was less than that of silver, which may be due to the yield of metal NPs obtained from the SP extract, and the total contents of carbon, oxygen, and copper varied between the synthesized AuNPs and AgNPs.

Consistent with literature reports [36], we observed that the average size of SP-AuNPs and SP-AgNPs was 5–30 nm and 10–40 nm, respectively. In a previous study, AuNPs obtained through a simple plant-mediated approach were highly monodispersed and spherical, with a size of 4 nm [37]. However, the shape and monodispersity of the NPs depend on the downstream preparation of synthesized NPs and biomolecule interactions with metal ions in the redox reaction [38]. *C. roseus* extract-mediated AuNPs and AgNPs showed elemental compositions of approximately 41 and 43 wt%, respectively [39]. In addition, EDAX spectra exhibited strong absorption signals for Au (at 1.5 keV) and Ag (at 3 keV); the reported peaks for *C. roseus* extract-mediated AuNPs and AgNPs are similar to our observations.

### 2.3. Transmission Electron Microscopy (TEM) of SP-AuNPs and SP-AgNPs

**Figure 3:**
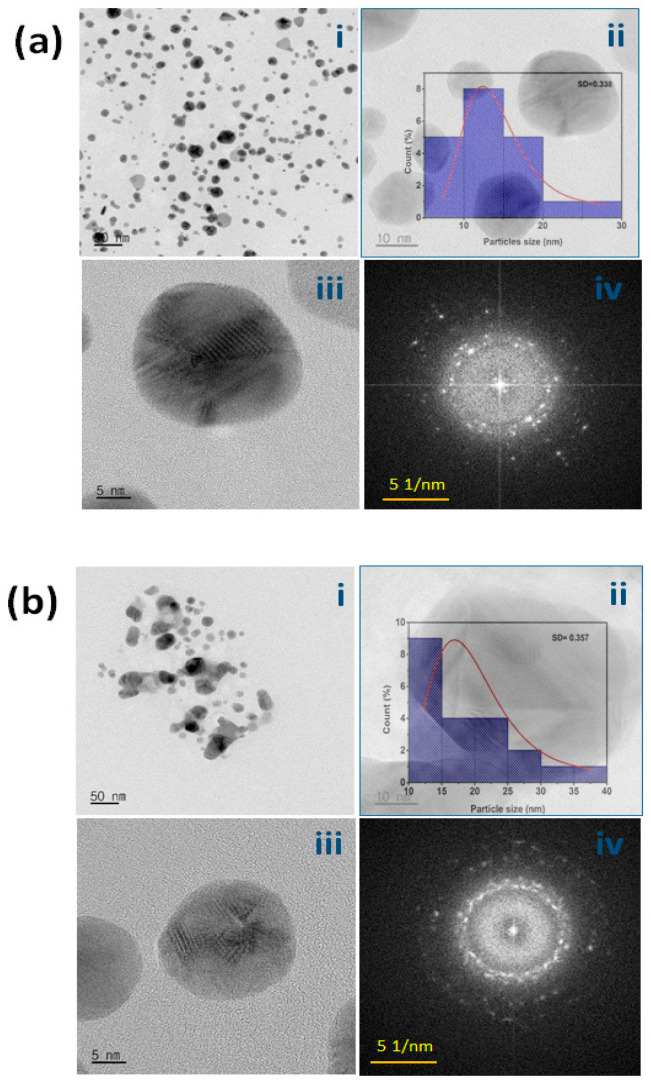
Transmission electron micrographs of SP-AuNPs and SP-AgNPs. (**a**) (**i**–**iii**) Shape and size of the synthesized AuNPs; scale = 50, 20, and 5 nm (inset: histogram of the particle size distribution of biosynthesized AuNPs analyzed using ImageJ). (**iv**) SAED pattern of AuNPs. (**b**) (**i**–**iii**) Shape and size of the synthesized AgNPs; scale bar = 50, 20, and 5 nm (inset: histogram of the particle size distribution of biosynthesized AgNPs analyzed using ImageJ). (**iv**) SAED pattern of AgNPs.

As shown in Figure 3a(i–iii), most SP-AuNPs were spherical and only a few were rod-shaped or triangular; the average size was 15–30 nm. Additionally, as shown in Figure 3b(i–iii), SP-AgNPs were spherical, with a uniform size range of 20–30 nm. SAED analysis showed that the synthesized SP-AuNPs and SP-AgNPs presented distinct ring patterns, indicating the presence of crystalline particles in the produced source (Figure 3a(iv),b(iv)). In addition, particle size distribution is presented as a histogram bar chart; SP-AuNPs and SP-AgNPs measured 27–31 nm on average and presented monodispersity, with adequate particle size distribution comparable to that of previously reported plant-derived NPs [40].

In a previous study, Qu et al. [41] described the morphology of plant-derived NPs; the NPs were uniformly dispersed in a colloidal solution and spherical, ranging in size from 5 to 9 nm. Meanwhile, in another study [41], *Limnophilia rugose*-derived metallic NPs (LR-MNPs) were nearly spherical and linked together to form clusters; a thin layer of biomolecules produced on the surface of the synthesized nanomaterials likely promoted cluster formation [42].

### 2.4. AFM Characterization of SP-AuNPs and SP-AgNPs

**Figure 4:**
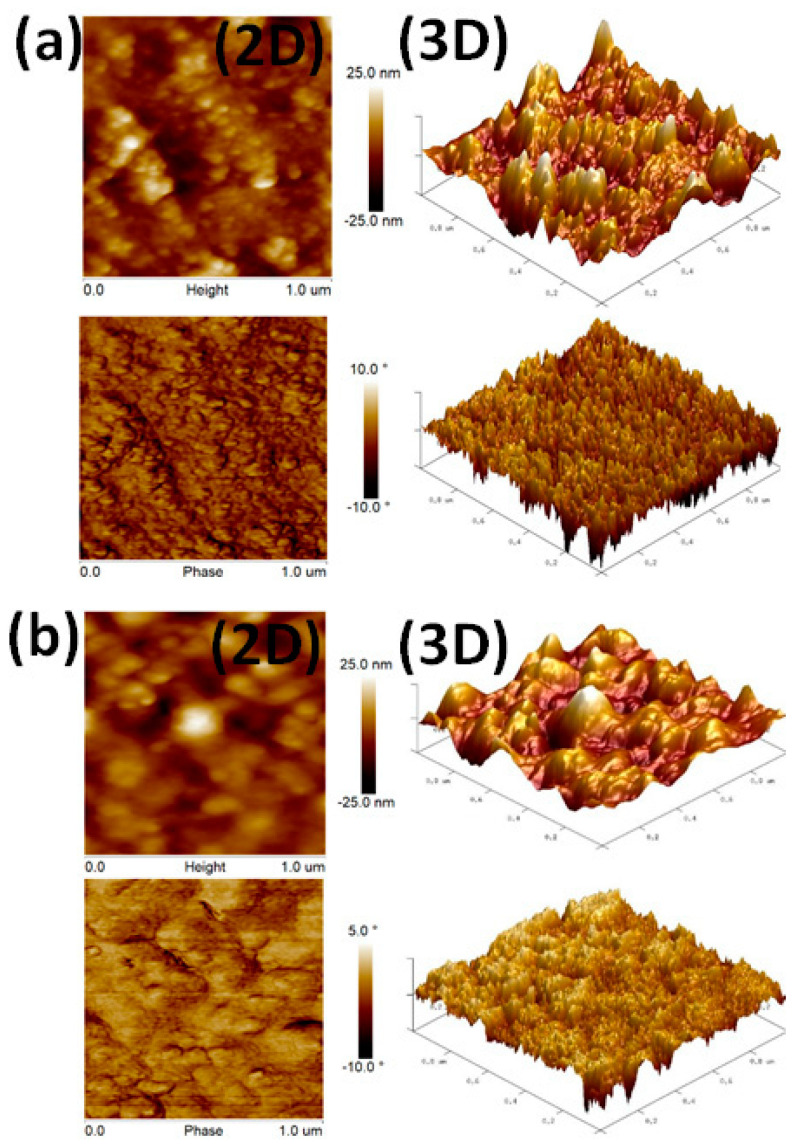
AFM structural analysis of biosynthesized AuNPs (**a**) and AgNPs (**b**) with 2D and 3D images of the height, surface phases, and size of nanoparticles.

SP-AuNPs and SP-AgNPs were subjected to AFM analysis, which provides 2D and 3D profiles of NP size, height, and phases. Figure 4a,b present the size and morphology of the synthesized AuNPs and AgNPs. Most of the produced SP-AuNPs and SP-AgNPs were spherical and only a few were of irregular shapes, forming little agglomerations in the samples. The molecular sizes of SP-AuNPs and SP-AgNPs were 15 and 40 nm, respectively. AFM data were highly correlated with TEM findings. Synthesized SS-AuNPs and SS-AgNPs (*Stereospermum suaveolens* (SS)) measured 12–18 and 20–40 nm, respectively, and showed a large surface area. Our AFM and TEM data were consistent with previous AFM findings [43].

### 2.5. FT-IR Spectroscopic Analysis of SP-AuNPs and SP-AgNPs

**Figure 5:**
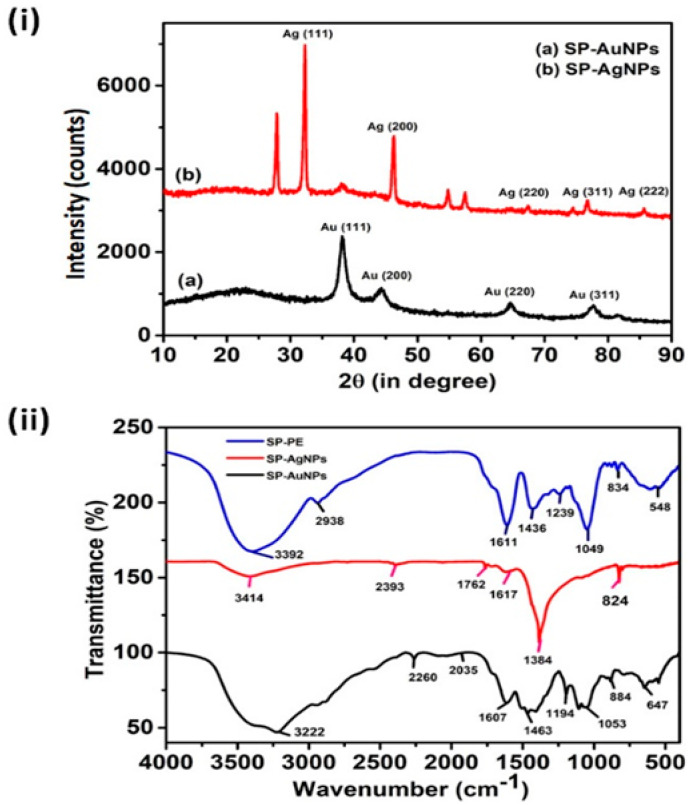
(**i**) Powder X-ray diffraction patterns of SP-AuNPs and SP-AgNPs. (**ii**) FT-IR spectra of SP-AuNPs, SP-AgNPs, and SP extract indicating functional groups in the plant extract and nanoparticle samples.

Figure 5i illustrates the FT-IR spectra of SP-AuNPs, SP-AgNPs, and SP extract. The FT-IR spectrum of the SP extract revealed a broad peak around 3392 cm^−1^, representing the stretching vibration of OH (hydroxyl), and a sharp narrow peak at 1650 cm^−1^, representing the vibration of C=C (carboxylic acids). The peaks at 2938 and 1611 cm^−1^ corresponded to C–H stretching (amide) and carbonyl derivatives, respectively. Peaks at 1436 and 1239 cm^−1^ corresponded to moderate stretching of the methyl (CH_3_) group. Meanwhile, bands at 1184 and 1049 cm^−1^ were related to the stretching vibration of the C–O group in ester and alcohol molecules, respectively. The absorption peak at 1239 cm^−1^ corresponded to the carbonyl functional groups (C=O) in the ester linkages of fatty acids and triacylglycerols in the SP extract. The sharp peak at 1049 cm^−1^ corresponded to the strong stretching vibration of C–O in alcohols, ethers, and esters. In the spectrum of the SP extract, regular peaks corresponding to O–H, N–H, and C–O derived from proteins, phenols, flavonoids, sugars, and tannins were noted, with slight deviations from peaks in spectra of SP-AuNPs and SP-AgNPs. The identified functional groups may be derived from bioactive metabolites, which are responsible for the reduction of metal salts to NPs and stability of the nanomaterial.

The FT-IR spectra of the SP extract and the biosynthesized SP-AuNPs and SP-AgNPs revealed significant similarities in terms of the characteristic peaks and intensities. However, in NP spectra, the peaks of certain organic moieties overlapped, and the intensity was higher than that in the SP extract spectrum. Furthermore, the broad band at 3270 cm^−1^ represented the stretching vibration of hydroxyl (-OH) groups in phenolic compounds, carboxylic acids, and terpenoids of the plant extract. Furthermore, the high-intensity peak at 1602 cm^−1^ was attributed to the C=C and C=O stretching vibration of aromatic and amino acid carbonyl groups [37]. The SP extract is rich in phenolic groups, which allows Au^3+^ and Ag^+^ to nucleate with Au^0^ and Ag^0^, respectively. In addition to phenolics, the SP extract contains an array of metabolites that work together to form nuclei and zerovalent Au and Ag NPs of optimum size and shape [44,45]. In a previous study, spectra of C-AuNPs and C-AgNPs derived from the *Cannabis sativa* extract showed intense bands at 3340, 3269, and 3280 cm^−1^ corresponding to O–H stretching; at 2899 and 2918 cm^−1^ corresponding to C–H stretching; at 1421 and 1415 cm^−1^ corresponding to CH_3_ and CH_2_ asymmetric deformation; and at 1019 and 1014 cm^−1^ corresponding to C–O stretching [33]. The synthesized SP-AuNPs exhibited strong signals corresponding to primary amines and carbonyl stretching in the amide linkages derived from amino acid residues and proteins, which are essential compounds for NP formation and agglomeration [45]. Sedum group succulent plants contain certain major metabolite groups, such as flavonoids, tannins, terpenoids, carbohydrates, and amino acids. In addition, SP extract has been used as an antiseptic, antibacterial, and diuretic in traditional medicine, indicating the functions of this plant in folk medicine [46].

### 2.6. Powder XRD Characterization of SP-AuNPs and SP-AgNPs

The XRD patterns of SP-AuNPs and SP-AgNPs are shown in Figure 5i. Four diffraction peaks at the 2θ values of 38.4°, 44.7°, 64.7°, and 77.3° were assigned to (111), (200), (220), and (311) planes, respectively, of the face-centered cubic (fcc) structure of metallic SP-AuNPs. These findings were compared with the Joint Committee on Powder Diffraction Standards (JCPDS) pattern file (JCPDS No. 04-0784) for the Au sample. Similarly, the peaks at the 2θ values of 38.1°, 44.4°, 64.5°, and 77.6° were assigned to (111), (200), (220), and (311) planes, respectively, of SP-AgNPs. The patterns were compared to the JCPDS file (04-0783) for the Ag sample. The fcc crystalline structure of SP-AgNPs was used to index all patterns. The intensity of (111), (220), and (311) planes, in particular, was relatively high, indicating that the predominant growth of the assembled AuNPs and AgNPs in the sample was low. Furthermore, the presence of unassigned peaks on the XRD graphs of gold and silver samples (33.45 and 56.78) indicates the crystalline growth of bio-organic compounds on the surface of the synthesized metallic NPs.

The fcc structures of the synthesized SP-AuNPs and SP-AgNPs with assigned standard planes were compared with literature reports. For instance, the X-ray diffractograms of AuNPs exhibited four intense peaks at 38.2°, 44.4°, 64.6°, and 77.6°, which were assigned to (111), (200), (220), and (311) planes, respectively. Meanwhile, AgNPs showed peaks at 38.2°, 44.3°, and 77.7°, which were assigned to (111), (200), and (311) planes [47]. These results indicate the fcc structures of the synthesized AuNPs and AgNPs. In a previous study [48], Au NPs showed four peaks at the 2θ values of 38.09°, 44.40°, 65.56°, and 77.60°, which were assigned to (111), (200), (220), and (310) lattice planes, respectively. For AgNPs, the XRD peaks at 38.19°, 44.34°, 64.48°, and 77.49° were assigned to (111), (200), (220), and (311) lattice planes, respectively; these values were identical those reported for standard silver metal.

### 2.7. Antibacterial Activity of SP-AuNPs and SP-AgNPs

**Figure 6:**
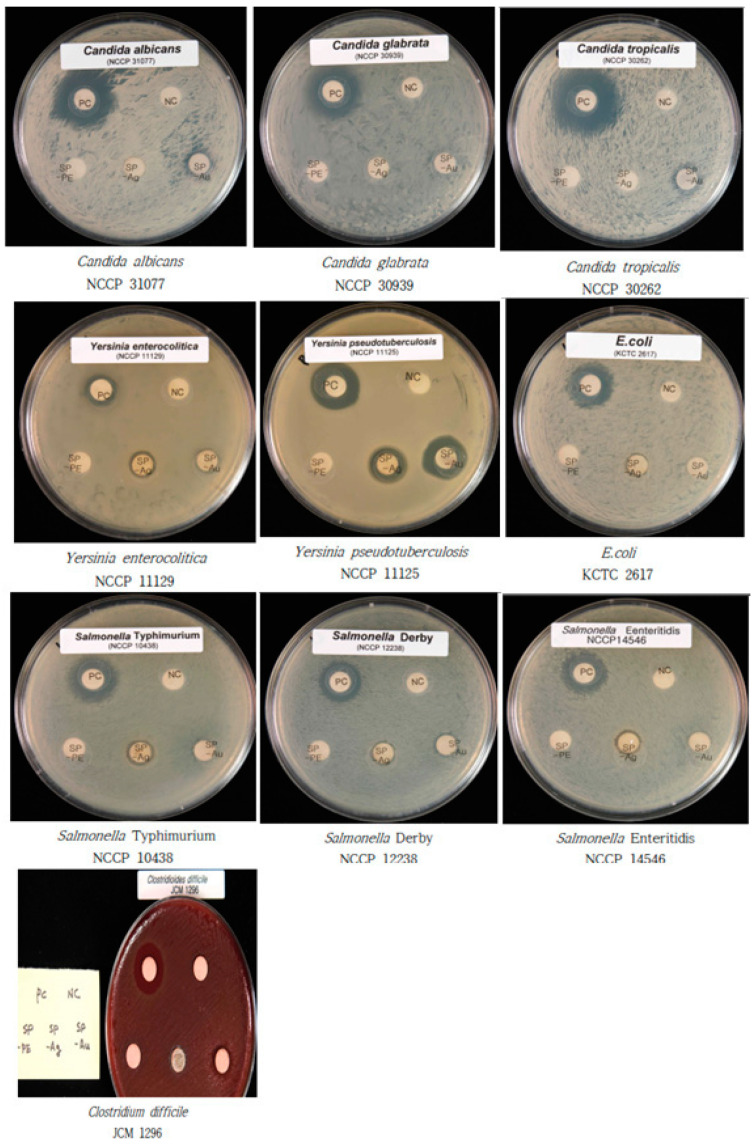
Antibacterial and antifungal activity of biosynthesized SP-AuNPs, SP-AgNPs, and SP extract analyzed using the agar disc diffusion assay. Zone of inhibition was observed and measured against Gram-negative bacterial, Gram-positive bacterial, and fungal livestock pathogenic strains.

The antibacterial activity of the SP extract, SP-AuNPs, and SP-AgNPs was evaluated using the agar disc diffusion method, and the results are shown in Figure 6. The agar disc diffusion assay demonstrated the SP-AuNPs and SP-AgNPs evidently inhibited the growth of the selected livestock pathogenic bacteria, such as *Escherichia coli* (Gram-negative), *Salmonella derby* (Gram-negative), *Salmonella enteritidis* (Gram-negative), *Salmonella typhi* (Gram-negative), *Yersinia enterocolitica* (Gram-negative), *Yersinia pseudotuberculosis* (Gram-negative), and *Clostridium difficile* (Gram-positive). Specifically, the SP-AgNPs showed excellent antibacterial activity against *E. coli*, *S. derby*, *S. enteritidis*, *S. typhi*, *Y. enterocolitica*, *Y. pseudotuberculosis*, and *C. difficile*. Among these, SP-AgNPs showed the largest antibacterial zone of inhibition (ZOI) against *Y. pseudotuberculosis* (14 ± 3 mm) and moderate antibacterial activity against other strains, such as *S. derby*, *S. enteritidis*, *S. typhi*, *E. coli*, and *Y. enterocolitica* (ZOI > 9 ± 3 mm). Furthermore, SP-AuNPs showed the largest ZOI against *Y. pseudotuberculosis* (20 ± 3 mm); moderate activity against *S. typhi* (ZOI = 11 ± 3 mm), followed by *S. derby* (ZOI = 11 ± 3 mm) and *E. coli* (ZOI = 11 ± 3 mm); weak antibacterial activity against *Y. enterocolitica* (ZOI < 9 mm); and no activity against *S. enteritidis* (Table 1). The bactericidal activity of SP-AuNPs and SP-AgNPs against livestock pathogens was much stronger than that of the SP extract alone, supporting previously published data on the bioactivity of plant extracts and metal NPs [49,50,51]. Furthermore, increasing drug (plant extract) concentration may effectively control resistant pathogenic strains, which did not respond to the concentration of 20 µL. Furthermore, the antibacterial activity of the synthesized NPs was realized through their attachment to the bacterial cell wall, which depends on the surface area, size, and shapes of NPs. Additionally, the available active metabolites on the surface of NPs for interaction with pathogens may be another mechanism for the antibacterial activity of NPs. Of note, the SP-AuNPs and SP-AgNPs were highly effective against the tested Gram-negative and Gram-positive bacteria.

Furthermore, at 20 µL volume (stock concentration = 5 mg·mL^−1^), AgNPs exhibited potent antibacterial activity against Gram-negative bacteria (*S. typhi, S. enteritidis, S. derby, E. coli, Y. enterocolitica*, and *Y. pseudotuberculosis*), with the largest ZOI against *Y. pseudotuberculosis* (14 mm). Meanwhile, at 20 µL concentration, AuNPs showed moderate antibacterial activity against Gram-positive and Gram-negative pathogenic bacteria (*S. derby*, *E. coli*, *Y. pseudotuberculosis*, and *C. difficile*). Overall, the antibacterial activity of AuNPs and AgNPs depends on the size, morphology, shape, and concentration of NPs and type of pathogens. As such, smaller NP size and unique shapes are more effective in inhibiting the bacteria because of increased interaction with bacterial cell membrane [52]. In a previous study, *Aloe vera* extract did not show inhibitory activity against bacterial strains, such as *E. coli*, *Pseudomonas aeruginosa*, and *Staphylococcus aureus*. In the present study, the synthesized AgNPs were more effective at higher concentrations (10 µg·mL^−1^) than at lower concentrations (5 µg·mL^−1^). The synthesized NPs were showed the maximum ZOIs against *Escherichia coli* (7 mm), followed by *Staphylococcus aureus* (4 mm) and *Pseudomonas aeruginosa* (4 mm). Metal silver NPs act as broad-spectrum antimicrobial agents against various human and animal pathogens. Specifically, *Aegle marmelos*-synthesized NPs showed antimicrobial activity against clinical pathogenic bacteria, such as *E. coli*, *S. aureus*, and *P. aeruginosa* [53,54]. In addition, plant extract-biosynthesized AgNPs showed moderate activity against *S. aureus*, while AgNPs alone showed slightly lower antibacterial activity against *E. coli* and *S. typhi* [39]. The key mechanism underlying the antibacterial activity of NPs is the electrostatic interactions between the metallic NPs and the negative charges on microbial cell wall, resulting the denaturation of proteins, increase in cell wall permeability, leakage of cell wall components, production of reactive oxygen species, and blockade of transportation channels toward cells death [55,56,57,58]. The minimum inhibitory concentrations (MICs) of SP-AuNPs, SP-AgNPs, and standard antibiotics against livestock pathogenic microorganisms were explored. The MIC of ampicillin ranged between 0.019 and 0.347 µg·mL^−1^ (Table S1). *Y. pseudotuberculosis* and *C. difficile* showed the highest susceptibility to AuNPs at MICs of 0.076 and 0.05 µg·mL^−1^, respectively. Similarly, the selected bacterial strains showed the highest susceptibility to AgNPs at the MIC of 0.102 µg·mL^−1^. Meanwhile, *S. enteritidis* and *C. glabrata* showed the highest resistance to AuNPs at higher concentration (0.250 µg·mL^−1^). Additionally, *Y. pseudotuberculosis* showed the highest susceptibility to AgNPs at the MIC of 0.50 µg·mL^−1^. The observed MICs for SP-AuNPs and SP-AgNPs were comparable to those for standard antibiotics, such as ampicillin, which is used to treat pathogenic microbial infections [59]. These findings confirm that the synthesized SP-AuNPs and SP-AgNPs possess antimicrobial potency against livestock pathogenic bacterial and fungal strains.

### 2.8. Antifungal Activity of SP-AuNPs and SP-AgNPs

Figure 6 shows the antifungal activity of SP-AuNPs and SP-AgNPs against *Candida* species. SP-AuNPs (20 µL, stock concentration = 5 mg·mL^−1^) showed moderate inhibitory activity against *Candida albicans* (9 ± 3 mm) and *Candida tropicalis* (9 ± 3 mm) but no activity against *Candida glabrata*. Interestingly, SP-AuNPs inhibited the growth of *C. albicans* and *C. tropicalis*. SP-AgNPs showed no effect against the three *Candida* strains. Moreover, the ZOIs of standard drugs against the three *Candida* species were larger than those of SP-AuNPs and SP-AgNPs.

Surprisingly, SP-AuNPs showed moderate antifungal activity against *C. albicans* and *C. tropicalis*. The larger surface area and smaller size of the synthesized SP-AuNPs may allow them to easily interact with the fungal cell membrane. Meanwhile, SP-AgNPs (20 µL, stock concentration = 5 mg·mL^−1^) did not show antifungal activity against the tested *Candida* strains. The fungicidal activities of SP-AuNPs and SP-AgNPs may be attributed to their physical interaction with the pathogen or induction of oxidative damage by NPs to the pathogens. AgNP treatment produced a specific response against *C. albicans* and *Saccharomyces cerevisiae* [10]. Moreover, Ag_2_O/AgNPs have been reported to be effective control agents against the growth of the three *Candida* species tested. In addition, the gold and silver NPs possess a higher affinity to protein, implying that the NPs tend to bind the membrane proteins of pathogenic cells [31]. According to the hard–soft acid–base theory, AuNPs and AgNPs showed a higher affinity to bind with the phosphorus and sulfur moieties of the cell membrane proteins of pathogenic organisms and inhibited cell replication.

### 2.9. Antioxidant Activity of SP-AuNPs and SP-AgNPs

Table 1 shows the DPPH free radical scavenging activity of the SP extract, SP-AuNPs, and SP-AgNPs. The DPPH free radical scavenging activity increased as the concentration of SP-AuNPs and SP-AgNPs increased. The free radical scavenging activity of SP-AuNPs and SP-AgNPs was 41% and 43%, respectively, at the stock concentration of 5 mg·mL^−1^. However, it was much lower than that of the ascorbic acid standard. The weak antioxidant activity of SP-AuNPs and SP-AgNPs, however, is consistent with earlier reports. For instance, *Plantago lanceolata* aqueous extract and AgNPs achieved peak antioxidant activity of 62% and 17% at the concentration of 100 µg·mL^−1^, respectively, indicating higher potential than the standard ascorbic acid (69% at 100 µg·mL^−1^) [60]. Moreover, *Mimosa tenuiflora* plant extract showed 50% activity, similar to other plant extracts. However, the antioxidant activity of standard drugs, such as vitamin C and catechin (46% and 60%, respectively), is higher than that of natural compounds [61].

### 2.10. Cytotoxicity of SP-AuNPs and SP-AgNPs

**Figure 7:**
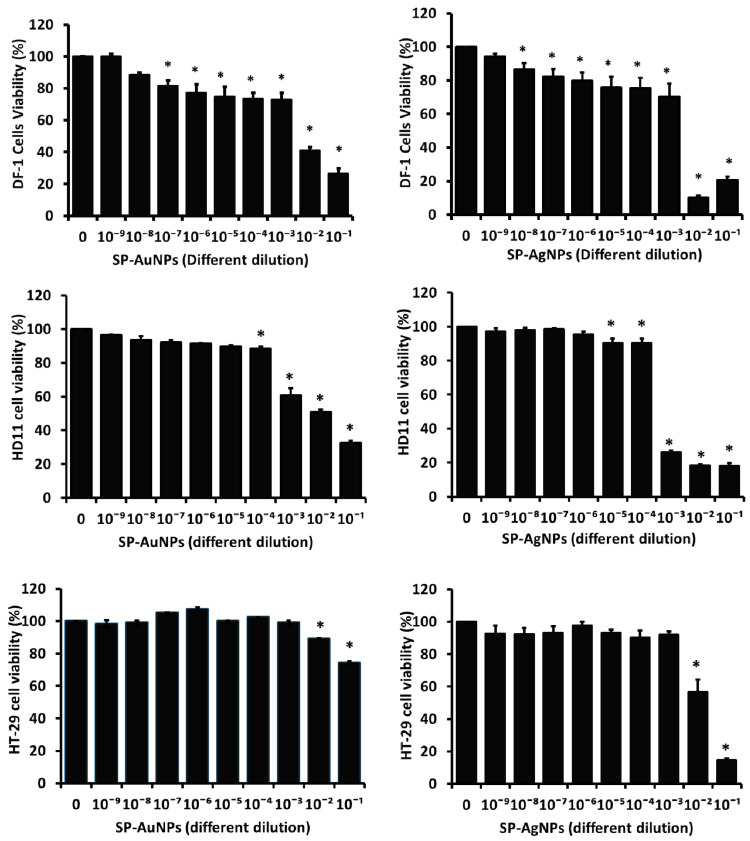
Cytotoxicity of SP-AuNPs and SP-AgNPs in chicken fibroblasts (DF-1), chicken macrophages (HD11), and human colon cancer cells (HT-29) at 24 h as determined using the WST-1 cell viability assay. Experiments were performed in triplicate. (*)−Values are presented as mean ± SE. *p* < 0.005 was considered statistically significant compared with the control, as calculated by one-way ANOVA and Duncan’s multiple post hoc test (. Stock concentration: 10 mg·mL^−1^; 0: control (no NPs), 10^−1^ dilution: 1 mg·mL^−1^; 10^−9^ dilution: 10 fg·mL^−1^.

At concentrations ranging from 10^−4^ to 10^−9^ (1 µg·mL^−1^–10 fg·mL^−1^), SP-AuNPs and SP-AgNPs exhibited low cytotoxicity against the cell lines used in the present study; however, at higher concentrations (10^−1^ to 10^−3^–1 mg·mL^−1^–10 µg·mL^−1^), excellent cytotoxicity was noted in both chicken and human cancer cell lines. Cell viability exceeded 85% when the two chicken cell lines and the human cancer cell line were cultured with 10^−4^ to 10^−9^ (1 µg·mL^−1^–10 fg·mL^−1^) concentrations of SP-AuNPs and SP-AgNPs. However, cell viability dropped below 30% after 24 h of incubation with higher concentrations (10^−1^ to 10^−3^–1 mg·mL^−1^–10 µg·mL^−1^) of SP-AuNPs and SP-AgNPs (Figure 7). Microscopic image analysis revealed the morphology of chicken and human cancer cells treated with SP-AuNPs and SP-AgNPs after 24 h (Figure S2).

At higher concentrations (10^−1^ (1 mg·mL^−1^) to 10^−3^ (10 µg·mL^−1^) dilution), both SP-AuNPs and SP-AgNPs did not affect the cell structure but slightly reduced percent cell viability. The maximum concentration of NPs that produced a significant impact on cell viability and morphology was determined (Figure S2). These results are consistent with previous reports. In a previous study, at 100 µg·mL^−1^, D-AuNPs did not affect cell viability after 48 h of treatment [62]. Meanwhile, AgNPs exhibited weak cytotoxicity in A549 cells at same concentration (100 µg·mL^−1^) [63]. In the present study, the cytotoxic effects of SP-AuNPs and SP-AgNPs on chicken and human cancer cells were weak. However, SP-AgNPs showed significant cytotoxicity (<50% cell viability) at the concentrations of 100 and 200 µg·mL^−1^. In particular, silver NPs showed stronger cytotoxicity against chicken macrophages than gold NPs at the same concentration [5,64]. Furthermore, D-AgNPs (*Dendropanax morbifera*) significantly inhibited cancer cell growth (>70%) after 48 h of exposure to higher concentrations of 100 µg·mL^−1^ [65]. The cytotoxicity of plant-derived NPs may involve the synergic effects of plant compounds. However, the cytotoxicity of these NPs may increase with increasing concentration. Another possible factor contributing to the cytotoxicity of these NPs is their tendency to aggregate in protein or aqueous media [66,67]. The aggregates of nanomaterial, which may adhere to the cell membranes and block cell function or even result in apoptotic cell death, may be the source of toxicity of metallic NPs [68].

## 3. Conclusions

Here, we synthesized AuNPs and AgNPs from the aqueous extract of *S. pink ruby* using green chemistry technology. The synthesized SP-AuNPs and SP-AgNPs ranged in size from 5 to 30 nm and from 10 to 45 nm, with average diameters of 25 and 50 nm, respectively. The metal composition of the NPs was determined based on EDX spectra, which indicated 25% Au and 67% Ag in the sample. In addition, other organic moieties, possibly derived from adherent biomolecules in the plant extract, were detected. FT-IR spectra revealed hydroxyl groups, amines, aldehydes, ketones, flavonols, and other phytochemicals responsible for the rapid reduction of metal ions present in the SP extract. Next, the antibacterial properties of SP-AuNPs and SP-AgNPs were examined; the NPs showed potent inhibitory activity against *E. coli, S. typhi, S. derby,* and *Y. pseudotuberculosis* but weak activity against *Candida* species (ZOI of <9 mm for AuNPs). Nonetheless, SP-AuNPs and SP-AgNPs may inhibit livestock pathogenic Gram-negative bacteria. Furthermore, we tested the cytotoxicity of SP-AuNPs and SP-AgNPs against avian-based cell lines, such as chicken fibroblasts and macrophages. The NPs showed no cytotoxicity at lower concentration, although higher concentrations significantly reduced cell viability in both cell lines. Therefore, the synthesized SP-AuNPs and SP-AgNPs may serve as potent antibacterial agents to control pathogenic infections in poultry and livestock animals. Furthermore, these NPs may serve as novel sources of antimicrobial agents to treat livestock infections, adjuvants for vaccine development, and biomedical applications in the near future.

## 4. Materials and Methods

### 4.1. Chemicals

Gold (III) chloride trihydrate (HAuCl_4_·3H_2_O, Cat. No. 520918), silver nitrate (AgNO_3_, Cat. No. 209139), NaOH, and HCl were purchased from Sigma Aldrich (Republic of Korea). All chemical reagents were of HPLC grade. Cell culture media and supplements were obtained from Thermo Fisher Scientific Korea (Seoul, Republic of Korea). The Center for Industrialization of Agricultural and Livestock Microorganism (CILAM, Jeongeup, Republic of Korea) provided pathogenic bacterial and fungal cultures.

### 4.2. Plant Collection and Preparation of Aqueous Extract

*S. pink ruby* was obtained from a local market in Republic of Korea and maintained at the Department of Biotechnology, Hoseo University, Republic of Korea. Fresh leaves (50 g, cut into 1–2 cm pieces) were mixed with 150 mL of sterile distilled water. The aqueous plant extract was prepared in an autoclave for 10 min at 110 °C under 39.23 kPa pressure. The extract was passed through a cellulose acetate filter (0.22 µm pore size; Sartorius, Göttingen, Germany) under sterile conditions and stored at 4 °C.

### 4.3. Biosynthesis of AuNPs and AgNPs

AuNPs and AgNPs were biosynthesized following a previously described method [69] with minor modifications. AuNPs were synthesized using 5 mL of aqueous SP extract and 45 mL of HAuCl_4_ (0.001 M) solution at 30 °C ± 2 without light exposure. The reaction mixture immediately turned pink, indicating the formation of NPs in the synthesis medium. The reaction mixture was then transferred to a hot plate at 37 °C for 30 min to complete the growth of AuNPs in a medium. AgNPs were synthesized from 5 mL of SP aqueous extract and 45 mL of AgNO_3_ (0.001 M) solution without light exposure. The mixture gradually turned from colorless to yellowish-brown over 15 min, indicating the formation of AgNPs in the colloidal solution. The appropriate reaction procedure was repeated two times. The biosynthesized NPs were then centrifuged at 10,000× *g* for 15 min, and the pellet was freeze dried overnight. Powdered NP samples were stored at 4 °C for further experiments. The biosynthesis procedure of AuNPs and AgNPs was optimized using various reaction parameters, such as pH (4, 5, 6, 7, and 8), plant extract ratio (1:2, 1:4, 1:6, 1:8, and 1:10), and metal salt concentration (10^−2^, 10^−3^, 10^−4^, 10^−5^), to achieve the maximum yield and quality. Further, the NP quality was preliminarily checked by assessing the appearance of color in the synthesis medium, and the UV–Vis absorbance spectra of the NP colloidal solution were analyzed.

### 4.4. Characterization of SP-AuNPs and SP-AgNPs

The UV–Vis absorption spectra of SP-AuNPs and SP-AgNPs were obtained using a UV–Vis spectrophotometer (UV 1800, Japan; 200–800 scan range and 1 nm resolution).

The size, structure, and elemental ratio/mapping of the NPs were determined using FESEM, EDX, and elemental mapping using an electron microscope (JEM 2100 F; JEOL, MA, Japan) operated at 200 kV. NP powder samples were sputter coated on a carbon film in a sample holder at 90 °C for 90 s. Then, the structure, morphology, and SAED patterns of the synthesized NPs were studied using a transmission electron microscope (Zeiss TEM Microscope, Carl Zeiss, Oberkochen, Germany). TEM specimens were prepared using carbon-coated copper grids. The prepared aqueous NP samples were deposited drop-wise onto the TEM and sample grids, which were dried at 80 °C for 10 min. Further, the grids were used for particle visualization using a transmission electron microscope. ImageJ was used to calculate the diameter of the NPs and their particle size distribution within the samples [70].

The crystalline nature of the freeze-dried AuNPs and AgNPs was evaluated using an X-ray spectrophotometer (D8 Advance, Bruker Corp., Billerica MA, USA) at room temperature with nickel-filtered Cu-Kα radiation at 1.54 Å operated at 40 kV voltage and 30 mA current. The XRD pattern ranges were recorded at 2θ from 5° to 90° with a scan rate of 1·min^−1^ and a slit width of 6.0 mm [71].

The functional groups of the synthesized AuNPs and AgNPs were characterized by FT-IR spectroscopy (PerkinElmer Inc., Waltham, MA, USA) using the KBr method in the matrix scan range of 4000–500 cm^−1^ and at the resolution of 4 cm^−1^ [72].

### 4.5. Antibacterial Activity of SP-AuNPs and SP-AgNPs

The antibacterial activity of the SP extract, SP-AuNPs, and SP-AgNPs was measured against livestock pathogenic microbes, including *Clostridium difficile* (JCM 1296), *Escherichia coli* (KCTC2617), *Salmonella derby* (NCCP 12238), *Salmonella enteritidis* (NCCP 14546), *Salmonella typhimurium* (NCCP 10438), *Yersinia enterocolitica* (NCCP 11129), and *Yersinia pseudotuberculosis* (NCCP 11125), using the disc diffusion method. For the assay, rich Luria broth agar, Sabouraud dextrose (SD) agar, and brain heart infusion agar plates were prepared. Next, 100 µL of bacterial suspension (~1.2 × 10^8^ CFU·mL^−1^) was spread on the solid agar plates. SP-AuNP, SP-AgNP, and SP extract samples were prepared and dissolved (20 µL each, stock concentration = 5 mg·mL^−1^) in a sterile 6 mm blank disc. On each disc, the NP solution was spotted and allowed to dry before the disc was placed on the agar plate and incubated at 37 °C for 24 h. Commercial antibiotics (ampicillin) were used as the positive control (20 µg). After incubation, the diameter of the ZOI around each disc was measured using a measuring scale. All experiments were performed in triplicate [73].

### 4.6. Antifungal Activity of SP-AuNPs and SP-AgNPs

The antifungal activity of the biosynthesized SP-AuNPs and SP-AgNPs as well as of the SP extract was tested against three pathogenic fungal strains: *Candida albicans* (NCCP 31077), *Candida glabrata* (NCCP 30939), and *Candida tropicalis* (NCCP 30262). The selected fungal pathogens were grown in PDA broth at 28 °C for 24 h using the disc diffusion technique. Fresh fungal cultures (100 µL each, i.e., ~2 × 10^5^ CFU·mL^−1^) were evenly spread in a sterile PD agar Petri plate. SP-AuNPs, SP-AgNPs, and SP plant extract (20 µL each, stock concentration = 5 mg·mL^−1^) were separately added to a 6 mm blank disc and incubated for 3 h to allow the complete adsorption of the samples. The discs were then dried and placed aseptically on fungal-inoculated Petri plates. The plates were incubated at 25 °C for 3 days. The antifungal activity of each sample was measured based on the diameter of the ZOI around each disc using a measuring scale.

### 4.7. MIC Study

The lowest concentration of AuNPs/AgNPs that completely inhibited the bacterial/fungal growth in the medium was considered the MIC. A loopful of pathogenic bacterial/fungal cultures was inoculated in individual wells containing MHA medium and treated with different concentrations of NPs (20, 10, 5, 2.5, and 1.25 µL samples; stock concentration = 5 mg·mL^−1^). The sample-treated wells showing no visual bacterial growth (at the lowest concentration) after 8–12 h of incubation were further spotted onto MHA agar medium and incubated at 37 °C for 24 h.

### 4.8. Antioxidant Activity of SP-AuNPs and SP-AgNPs

The antioxidant activity of SP-AuNPs and SP-AgNPs was analyzed using the 2,2-diphenyl1-picrylhydrazyl (DPPH) free radical scavenging assay, as described in a previous study [74] with minor modifications. Briefly, 10 mL of 0.2 mM DPPH stock solution was prepared in methanol. Then, 100 µL aliquots of aqueous AuNPs/AgNPs (10^−1^, 10^−2^, 10^−3^, 10^−4^, and 10^−5^ dilutions, stock concentration = 5 mg·mL^−1^), plant extract, and standard solution (10–100 µg·mL^−1^) were mixed with 100 µL of methanolic DPPH solution. DPPH with methanolic solution alone was used as the control. All solutions were incubated in the dark at room temperature for 30 min. The DPPH antioxidant activity of each sample was determined based on optical density (OD) measured at 517 nm. Percent DPPH scavenging activity was calculated using the following equation:

DPPH free radical scavenging activity (%) = ([OD_control_ − OD_sample_])/[(OD_control_)] × 100

where OD_sample_ is the OD value of AuNPs, AgNPs, and plant extract/standard, and OD_control_ is the OD value of DPPH methanolic solution (control).

### 4.9. Cytotoxicity of SP-AuNPs and SP-AgNPs

The cytotoxicity of SP-AuNPs and SP-AgNPs was determined using the WST-1 cell proliferation and viability assay. Chicken (DF-1 cells: chicken embryonic fibroblast cells, HD11 cells: chicken macrophage cells) and human (HT-29: human colon cancer cells) cells were cultured in Dulbecco’s modified Eagle’s medium (DMEM) supplemented with 10% fetal bovine serum (FBS) and 1% penicillin–streptomycin (Gibco, Gaithersburg, MD, USA). The cells were maintained at 37 °C in a humidified atmosphere with 5% CO_2_. The cells were seeded in a 96-well plate at the density of 1 × 10^5^ cells·well^−1^ and incubated for 24 h to reach 90% confluence. After reaching confluence, the cells were treated with different concentrations of SP-AuNPs and SP-AgNPs (10^−1^, 10^−2^, 10^−3^, 10^−4^, and 10^−5^ dilutions, stock concentration = 10 mg·mL^−1^) for 24 h. Then, 10 µL of WST-1 solution was added to each well, and the plates were incubated for an additional hour [75]. Then, the OD of each plate was measured at 490 nm using a microplate reader (Thermo Fisher Scientific Solutions Co., Ltd., Waltham, MA, USA). Percent cell viability was calculated using the following equation:

Cell viability (%) = (OD490_sample_ − OD490_blank_)/(OD490_control_ − OD490_blank_) × 100

### 4.10. Statistical Analysis

All experiments were performed in triplicate. Data are presented as mean ± standard error (SE). One-way analysis of variance (ANOVA) was used to determine significant differences in means among the samples, followed by Duncan’s multiple comparison test (SPSS 20.0, IBM, Armonk, NY, USA). *p* < 0.05 was considered significant; in figures and tables, significance is indicated with subscript lowercase letters (a, b, c, d, and so on).

## Figures and Tables

**Table 1:** Antibacterial activity of SP-AuNPs, SP-AgNPs, and SP extract against Gram-negative and Gram-positive pathogenic bacteria of livestock and free radical scavenging activity of the synthesized AuNPs and AgNPs as calculated using the DPPH assay. For the antimicrobial test, 20 µL volume of each NP suspension was prepared from 5 mg·mL^−1^ of stock concentration; for the antioxidant assay, 10^−1^–10^−5^ dilution was prepared from 5 mg·mL^−1^ of stock concentration.

Sr. No	Components	Antibacterial Activity in the Zone of Inhibition (mm)							Antifungal Activity			Antioxidant Activity
		ST	SE	SD	EC	YE	YS	CD	CA	CT	CG	DPPH (%)
1	SP extract	−	−	−	−	−	−	−	−	−	−	38
2	SP-AuNPs	+	−	+	w	w	+++	++	+	+	−	41
3	SP-AgNPs	+	+	+	+	+	++	−	−	−	−	43

+++, ≥14–20 mm; ++, ≥12–14 mm; +, ≥9 mm; w (weak), <9 mm; −, no inhibition; Abbreviations: ST: *Salmonella typhi*, SE: *Salmonella enteritidis*, SD: *Salmonella derby*, EC: *Escherichia coli*, YE: *Yersinia enterocolitica*, YP: *Yersinia pseudotuberculosis*, CD: *Clostridium difficile*, CA: *Candida albicans*, CT: *Candida tropicalis*, and CG: *Candida glabrata*.

## Data Availability

Data are contained within the article and Supplementary material.

## Supplementary Materials

The following supporting information can be downloaded at: https://www.mdpi.com/article/10.3390/antibiotics12030507/s1, Figure S1: UV−Vis absorbance spectra of biosynthesized SP-AuNPs and SP-AgNPs under different reaction parameters (pH, plant extract ratio, and metal salt concentration); Figure S2: Cells were treated with different concentrations of SP-AuNPs and SP-AgNPs (10^−1^ to 10^−9^ dilution) for 24 h. Morphological changes were observed in NP-treated and control cells using light microscopy (magnification = ×100); Table S1: MICs of SP-AuNPs, SP-AgNPs, and ampicillin against selected pathogenic microorganisms.
