# Supplementary material for: Sedeveria pink ruby Extract-Mediated Synthesis of Gold and Silver Nanoparticles and Their Bioactivity against Livestock Pathogens and in Different Cell Lines
Source: Antibiotics (Basel). 2023 Mar 3;12(3):507. doi: 10.3390/antibiotics12030507 (PMC10044096; doi:10.3390/antibiotics12030507)
Supplement: Supplementary file 1 [file antibiotics-12-00507-s001.zip › antibiotics-2170034-SI.pdf]

Supplementary information

Sedeveria pink ruby Extract-Mediated Synthesis of Gold and Silver Nanoparticles and Their Bioactivity against Livestock Pathogens and in Different Cell Lines

Palaniselvam Kuppusamy [1], Sujung Kim [1], Sung-Jo Kim [2], Myunghum Park [3,*] and Ki-Duk Song [1,*]

[1] Department of Agricultural Convergence Technology, Jeonbuk National University, Jeonju 54896, Republic of Korea; kpalaselvam@jbnu.ac.kr (P.K.); feelksj00@naver.com (S.K.)
[2] Division of Cosmetics and Biotechnology, College of Life and Health Sciences, Hoseo University,
Asan 31499, Republic of Korea; sungjo@hoseo.edu
[3] Research and Development Center, T&T Research, Anyang 14059, Republic of Korea
* Correspondence: pmh@tntresearch.co.kr (M.P.); kiduk.song@jbnu.ac.kr (K.-D.S.); Tel.: +82-31-689-3610 (M.H.P.); +82-63-219-5523 (K.-D.S.)

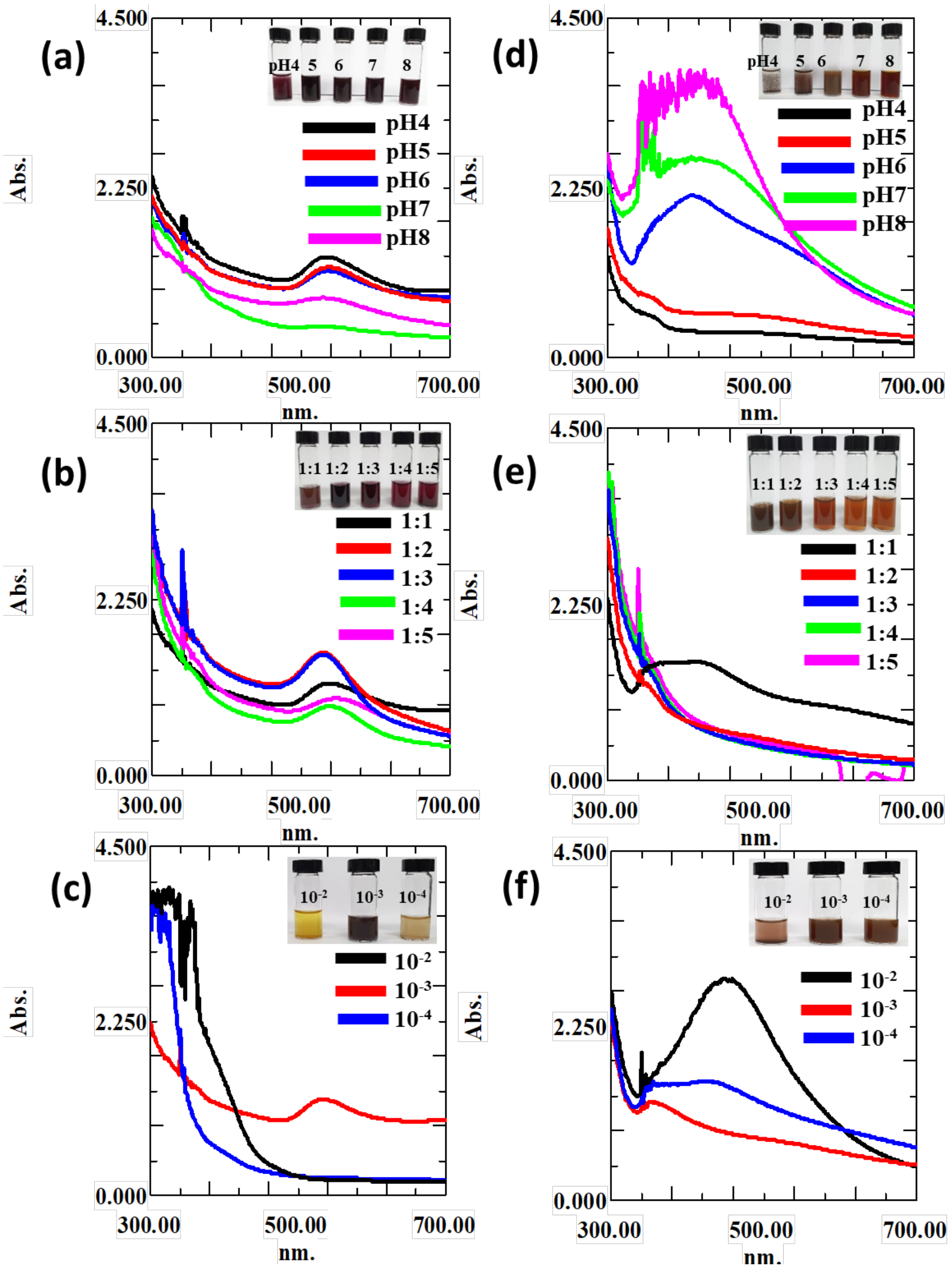

Figure S1. UV–vis absorbance spectra of biosynthesized SP-AuNPs and SP-AgNPs were optimized with different reaction parameters (pH, plant extract ratio and metal salt concentration). **(a-c)** UV–vis spectra of SP-AuNPs with different optimized ranges of pH, plant extract and metal salt concentration. **(d-f)** UV–vis spectra for SP-AgNPs prepared with different optimized ranges of pH, plant extract and metal ion concentration for 24 h.

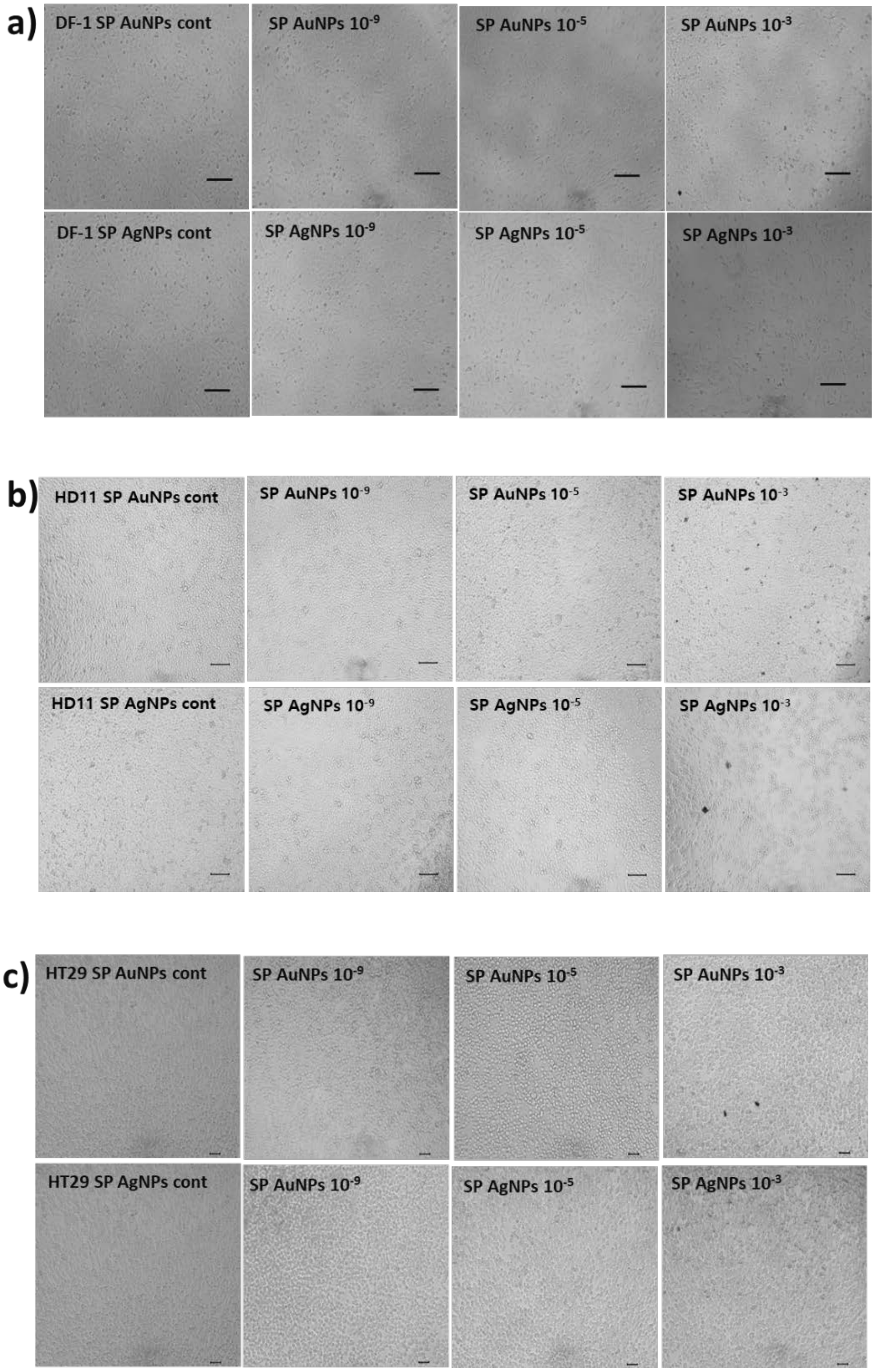

Figure S2. Cells was treated with different concentration of SP-AuNPs and SP-AgNPs (10^{-1} to 10^{-9} dilution) for 24 h. Morphological changes were observed in the nanoparticles treated and control cells by using light microscope (scale bar = x100). **(a)** DF-1 cells **(b)** HD11 cells and **(c)** HT-29 cells.

Table S1: MIC of *S.pink ruby* biosynthesized SP-AuNPs and SP-AgNPs against selected pathogenic microorganisms.

**Pathogens**	**MIC (μg.ml^{-1})**		
	**SP-AuNPs**	**SP-AgNPs**	**Control***
Salmonella typhi	0.102	0.102	0.347
Salmonella enteritidis	-	0.102	0.347
Salmonella derby	0.102	0.102	0.347
E. coli	0.102	0.102	0.039
Yersinia enterocolitica	0.102	0.102	NA
Yersinia pseudotuberculosis	0.076	0.050	NA
Clostridium difficile	0.050	-	0.019
Candida albicans	0.102	-	0.156
Candida tropicalis	0.102	-	0.156
Candida glabrata	-	-	0.156

*Ampicillin was used as standard antibiotic, **-** No activity.
